# RT-LAMP as Diagnostic Tool for Influenza—A Virus Detection in Swine

**DOI:** 10.3390/vetsci10030220

**Published:** 2023-03-13

**Authors:** Suzanna M. Storms, Joanna Shisler, Thanh H. Nguyen, Federico A. Zuckermann, James F. Lowe

**Affiliations:** 1 Department of Veterinary Clinical Medicine, University of Illinois at Urbana-Champaign, Urbana, IL 61802, USA; 2 Department of Microbiology, University of Illinois at Urbana-Champaign, Urbana, IL 61802, USA; 3 Department of Civil Engineering, University of Illinois at Urbana-Champaign, Urbana, IL 61802, USA; 4 Department of Pathobiology, University of Illinois at Urbana-Champaign, Urbana, IL 61802, USA

**Keywords:** LAMP, swine influenza, swine respiratory disease, veterinary diagnostics, point-of-care diagnostics

## Abstract

**Simple Summary:**

Rapid diagnostics for viral disease in livestock are urgently needed, especially for those that pose threats to human health and/or food security. Influenza A virus is a zoonotic disease that has a large reservoir in commercial swine herds. Reverse transcription loop-mediated isothermal amplification (RT-LAMP) is a tool that has the potential to detect influenza A virus in easy-to-obtain swine nasal samples in a short time period, with minimal laboratory equipment. This article outlines how to process nasal samples and complete an RT-LAMP assay in a laboratory setting, demonstrating a protocol that goes from sample collection to positive test results in less than one hour. Ultimately, this diagnostic test system can be developed for producers to identify sick animals on-farm, and to avoid logistical issues and time delays associated with the use of diagnostic laboratories. In addition, rapid results allow producers to implement interventions in real time, thus enhancing the efficacy of preventative measures and mitigating outbreaks.

**Abstract:**

Point-of-care diagnostic technologies are becoming more widely available for production species. Here, we describe the application of reverse transcription loop-mediated isothermal amplification (RT-LAMP) to detect the matrix (M) gene of influenza A virus in swine (IAV-S). M-specific LAMP primers were designed based on M gene sequences from IAV-S isolated in the USA between 2017 and 2020. The LAMP assay was incubated at 65 °C for 30 min, with the fluorescent signal read every 20 s. The assay’s limit of detection (LOD) was 20 M gene copies for direct LAMP of the matrix gene standard, and 100 M gene copies when using spiked extraction kits. The LOD was 1000 M genes when using cell culture samples. Detection in clinical samples showed a sensitivity of 94.3% and a specificity of 94.9%. These results show that the influenza M gene RT-LAMP assay can detect the presence of IAV in research laboratory conditions. With the appropriate fluorescent reader and heat block, the assay could be quickly validated as a low-cost, rapid, IAV-S screening tool for use on farms or in clinical diagnostic labs.

## 1. Introduction

Influenza A virus (IAV) is a serious threat to humans and livestock species in the United States and around the world. Human infections in the United States can be upwards of 31 million symptomatic illnesses per influenza season, with 140,000–700,000 influenza-associated hospitalizations per season between 2010–2020 [1]. IAV is zoonotic and able to transmit among many domesticated and wildlife species, and intensified agricultural practices for raising equine, avian, and swine species create potential reservoirs for variant viruses, increasing the threat of emergence [2]. Its persistence in human, livestock, and wildlife populations, as well as in the environment, enables continuous opportunities for reinfection, evolution, and immune escape [3,4,5].

IAV is a negative-sense RNA virus, consisting of eight genomic segments and classified by the hemagglutinin (H) and neuraminidase (N) genes. It is prone to random mutation, yielding from 1.8 × 10^−4^ to 2.5 × 10^−4^ base substitutions per virion, resulting in populations existing as quasispecies [6,7,8]. Equine and avian IAVs preferentially bind to α2-3 galactose-linked sialic acid, while human IAVs preferentially bind via an α2-6 galactose linkage on the sialic acid [9]. Swine represent a unique player in the world of influenza; they are considered a mixing vessel of avian, human, and swine strains due the presence of both of α2-3 and α2-6 sialic acids dispersed along the respiratory tract [10,11]. This allows for the generation of novel IAV strains through reassortment and further supports the need for rapid, pen-side diagnostics.

The COVID-19 pandemic brought attention to the need for rapid, point-of-care diagnostic testing and the challenges presented by limited reagent ability and lengthy assay turnaround time [12,13]. PCR and other nucleic acid amplification tests (NAATs), while faster than cell culture-based diagnostic tests, are difficult to implement in low-resource settings due to the cost of the equipment and lack of skilled staff to perform the assays. [14,15].

Loop-mediated isothermal amplification (LAMP) is a method of nucleic acid amplification used as a diagnostic system for microorganisms [16,17]. LAMP is a one-step nucleic acid amplification reaction that targets DNA sequences with equivalent sensitivity and specificity to RT-qPCR at an isothermal temperature [18]. Four to six separate regions within the DNA sequence are targeted, with four to six primers used in each reaction [19]. A reverse transcriptase enzyme simultaneously transcribes DNA from RNA sequences, while a strand displacing DNA polymerase amplifies the target sequences [20,21,22,23].

LAMP has also been used to detect the IAV matrix (M) gene in swine samples [24]. However, these publications tested LAMP-specific primers only on a narrow set of influenza virus subtypes, and did not account for M gene variation, which are increasing in prevalence in human isolates [25]. To overcome this shortcoming, our experiments began by developing a swine M gene primer set based upon strains isolated in the United States and testing their limit-of-detection using synthetic M genes. After initial screening and optimization, we assayed the efficacy of these primers to detect the M gene from nine different USA IAV-S (influenza A virus swine) strains that were chosen specifically for clade variation and recency of isolation. Finally, we show that these M primers also detect IAV collected from nasal samples from swine pigs at local barns.

Here, we describe the application of reverse transcription loop-mediated isothermal amplification (RT-LAMP) to detect the purified matrix gene of influenza A virus in swine (IAV-S) in nasal samples from swine. This study is the first step towards establishing a low-cost, pen-side test for IAV-S identification, and such a test would allow faster and more affordable strategies to be developed. Further application of this assay to enhance sample testing on-farm will be a great step forward for influenza detection in commercial settings, ultimately helping prevent IAV-S transmission.

## 2. Materials and Methods

### 2.1. Primer and gBlock DNA Design

All whole-genome-sequenced USA IAV-S isolated between 2017 and 2020 were included in the initial primer development. Matrix (M) genes (825 sequences), that were uploaded to GenBank, were obtained through the Influenza Research Database (IRD) through the web site at http://www.fludb.org (accessed on 1 February 2021), and were included in the analysis [26]. This timeframe was chosen to ensure that the primers designed would be able to detect current circulating strains in the United States. Sequences were used to create a consensus M gene sequence in Jalview, using MAFFT alignment [27]. The aligned FASTA sequences can be found in the Supplementary Materials. The LAMP primer sets were created using LAMP Designer software, version 1.1 (OptiGene, Horsham, UK) and were synthesized by Integrated DNA Technologies (manuf., Coralville, IA, USA), with the FIP and BIP primers undergoing HPLC purification, while the other four primers were purified by standard desalting [28].

Three primer sets were tested for the detection of a synthetic M gene and A/California/07/2009 H1N1 IAV grown in cell culture. All primer sequences were checked for self-complementarity and heterodimerization using Thermo Fisher’s Multiple Primer Analyzer [29]. Each set was evaluated and the set with the fastest time of amplification was selected for optimization. Optimization included serial dilutions of primer concentrations, and the set with the fastest concentration with the least amount of non-specific amplification is included in Table 1.

The consensus M gene was synthesized by Integrated DNA Technologies (Coralville, IA, USA) as gBlock DNA [30]. The gBlock DNA was diluted in 10-fold increments using DEPC-treated water and for standard curve generation, and used subsequently as positive controls.

Next, the F3/B3 primer set in Table 1 was screened for specificity in silico using Primer-BLAST [31]; 36,613 hits were realized. Of these 36,613 hits, the first 1006 were available for evaluation, and of those 1006 hits, 974 matched IAV-S isolates, and the remaining 22 matched IAVs isolated from humans outside of the USA.

### 2.2. RT-qPCR

RT-qPCR was used for validation of the RT-LAMP method. The Invitrogen SuperScript III Platinum SYBR green one-step kit from Applied Biosciences (Waltham, MA, USA) was used according to the manufacturer’s instructions, for the comparison of synthetic gBlock DNA with LAMP. The complete methods are found in the Supplementary Materials. Briefly, per 50 μL reaction, 4.75 μL of extracted RNA was added per well, and 45.25 μL of freshly prepared master mix was added per well. The primers and RT-PCR conditions used are those published in Annex 1, protocol 1, of the WHO information for the molecular detection of influenza virus handbook and target the M gene: M30F2/08 fwd 5′-ATGAGYCTTYTAACCGAGGTCGAAACG-3′, M264R3/08 rev 5′-TGGACAAANCGTCTACGCTGCAG-3′ [32]. PCR master mix was prepared immediately before use in a dedicated workspace with separate pipettes and filter tips to prevent contamination. The RT-qPCR reactions took place in a QuantStudio 3 machine (Applied Biosystems, Waltham, MA, USA). Reverse transcription for 30 min at 50 °C, initial denaturation for 5 min at 95 °C, and subsequent 40 amplification cycles were performed at 94 °C for 30 s, 50 °C for 30 s, and extension at 72 °C for 1 min. Melting curve analysis was performed per machine settings. Computed cycle threshold (Cq) was determined by the fluorescence signal of amplification above the background fluorescence. Performance of the RT-qPCR was validated using a positive control for the M gene in which serial 10-fold dilutions of gBlock DNA were used to establish a standard curve.

Clinical samples were screened using the USDA-licensed VetMAX™-Gold SIV Detection kit (Thermo Fisher Scientific, Waltham, MA, USA) according to manufacturer’s instructions. Complete methods are in the Supplementary Materials. A Xeno extraction and amplification control was included in each reaction tube to account for extraction yield and polymerase function. Results were normalized by using the 5% of the positive control ΔRn as the threshold for the clinical samples, as outlined in the manufacturer’s instructions. Positive and negative clinical sample RNA extracts were then used to validate the RT-LAMP reaction, and were the basis of the true positive and negatives during statistical analysis. All reactions and analyses were performed in the QuantStudio Design and Analysis software version 1.5.2 (Applied Biosystems, Waltham, NJ, USA).

### 2.3. Fluorescent RT-LAMP

The WarmStart LAMP 2x Master Mix fluorescent kit (New England Biolabs, Ipswich, MA, USA) was used with a modified protocol for all LAMP reactions. A total reaction volume of 12.5 μL was used, as previously published by Klein et al. [33]. Each reaction used 6.25 μL WarmStart 2x LAMP mix, 1.25 μL 10x LAMP primers targeting the M gene, and 0.25 μL of 50x fluorescent dye (NEB B1700S). An amount of 7.75 μL of the mix was placed in a 96-well PCR plate, and 4.75 μL of extracted RNA or gBlock DNA was added to each well and sealed with an optical film cover (Applied Biosystems). The LAMP assay was placed in a real-time qPCR thermocycler (QuantStudio 3, Applied Biosystems) and incubated at 65 °C, for 100 cycles, with 20 s for each cycle (total ~35.3 min). Fluorescence was detected at the end of each 20 s cycle. The time of amplification (TOA) was determined based on the computed fluorescence threshold, where the amplification reaction was above the background fluorescence. Samples that showed no amplification after 100 cycles or were amplified after the negative control sample were considered negative.

The fluorescent assay kit was chosen in order to provide more precise measurements (every 20 s) than are afforded by a colorimetric readout (read after 30 min, if not positive, extend reaction 10 min more), as the tubes need to be removed from the heat source for evaluation, resulting in inconsistent incubation temperatures [34]. We also found that the colorimetric assay, which is pH based, was very sensitive to elution buffers.

Clinical samples were screened after initial assay validation. RNA extracts were screened as IAV positive or negative via RT-PCR, and the same RNA extract was tested immediately following RT-PCR screening to determine concordance with the RT-LAMP assay and primers. Samples with PCR Cq values between 14 and 40 were used for the comparative PCR and LAMP assays.

### 2.4. Laboratory Virus Culture and Extraction

The assay was developed using the A/California/07/2009 H1N1 virus grown on MDCK cells kindly provided by the University of Illinois Veterinary Diagnostic Laboratory. Cells were maintained in minimal essential medium (MEM) (Corning) supplemented with 10% newborn calf serum (Gibco), 1% Anti-Anti (Gibco), at 37 °C/5% CO_2_. Viral stocks were generated by a single passage in cells, following the protocol of Balish et al., 2013 [35]. Briefly, viral stocks were diluted in MEM and allowed to bind to confluent monolayers of cells for 1 h at 37 °C/5% CO_2_. The media were then removed and replaced with viral culture media, supplemented with influenza virus growth medium containing complete Dulbecco’s MEM (Corning) with 7.5% BSA, and 2 μg/mL TPCK-trypsin (Worthington). TCID50 of 10 × 7^4^/mL of virus was used in each assay, calculated according to the Reed–Muench method [36]. After initial assay development, nine additional whole-genome-sequenced swine-isolated IAVs of different subtypes belonging to different clades were chosen and obtained from the NVSL swine influenza repository. The viruses were chosen based upon the currently circulating viruses (>1894 isolates) that were submitted to the Iowa State University Veterinary Diagnostic Laboratory and subtyped from 2017 to 2020, as displayed in their Heat Map tool. Only viruses that were whole-genome sequences were chosen for analysis. These viruses were grown in the lab in the same manner as above and were processed for use in the assay. The cultured viruses and their HA and NA diversity are shown in Table 2.

RNA was isolated from cell culture-grown viruses using the Cytiva Sera-Xtracta Virus/Pathogen kit (Marlborough, MA, USA) according to the manufacturer’s instructions. 140 μL of clarified viral supernatant was used per extraction and an elution volume of 100 μL was used. Detailed methods of extraction can be found in the Supplementary Materials, Section S2.

### 2.5. Clinical Sample Collection and Processing

All sampling methods and animal use protocols were conducted in compliance with the University of Illinois Institutional Animal Care and Use Committee (No. 19199) and the Institutional Biosafety Committee guidelines. All samples were handled and processed in a Biosafety Level 2 laboratory. Samples were collected from four swine herds in Illinois, USA. Weaned pigs, from 3 to 13 weeks of age, were restrained manually or with a snare for sample collection. An aluminum-handled mini-tipped swab (Puritan) was inserted into each nares then placed into a sterile tube containing 500 μL of BHI (brain–heart infusion) viral transport medium containing penicillin G and streptomycin sulfate [37,38]. All samples were kept on ice for transport to the lab, followed by long-term storage at −80 °C. Samples were thawed and 140 μL of sample was used for RNA extraction, as mentioned in Section 2.4.

### 2.6. Statistical Analysis

Results were analyzed using receiver operating characteristic (ROC) analysis, which is a useful tool for evaluating the accuracy of binary results, and often used for laboratory testing [39]. The RT-LAMP results were classified into two categories, true positive and true negative, based upon paired PCR screening. ROC curves were calculated using the GraphPad Prism software package, version 9.4.1 (GraphPad Software, LLC, San Diego, CA, USA) and Origin(Pro), Version 2023 (OriginLab Corporation, Northampton, MA, USA). This rating method, represented as the area under the ROC curve, is the probability that a random pair of positive and negative samples will be correctly stratified into their respective group based upon a specified threshold. Thresholds are created by comparing the average minutes-to-positive of the samples and the midway point between the positive and negative sample values. Sensitivity was calculated as the percentage of samples that were positive before the threshold time. Specificity is calculated as the percentage of the negative samples that were detected after the threshold time, or never detected. The ROC curves for the gBlock DNA and clinical samples are displayed in Supplementary Materials, Figure S1a.

## 3. Results

### 3.1. Fluorescent RT-LAMP Limit of Detection in Syntethic DNA

To determine the limit of detection (LOD) of the fluorescent RT-LAMP for the consensus M gene, the synthetic gBlock DNA was measured in a series of 10-fold dilutions to determine copy number sensitivity. gBlock DNA samples were serially diluted from 10^9^ to 10^1^ copies per reaction, with 10^6^ to 10^1^ copies tested in five technical replicates. These were added to reactions and these samples were positive controls. There were reactions prepared in parallel that did not receive gBlock DNA and these were considered to be negative controls. In total, 142 positive samples (containing gBlock DNA) and 126 negative samples (did not contain gBlock DNA) were used to determine the sensitivity and specificity of the assay. The time of amplification of all 268 samples was used to determine the optimal threshold time for the assay. We used OriginLab Pro software version 10.0.0.154, to calculate the average time of amplification between a positive sample and the average time to amplification of the negative samples. All samples were stopped on or before 100 cycles, or at 35.3 min.

The sensitivity was determined by calculating how many of the samples spiked with the synthetic gBlock DNA were detected at specific time points (true positive detection), and the specificity calculated as 1 is the number of negative samples also detected at the same time points (false positive). In this way, the program displays the sensitivity and specificity of the assay at different thresholds. The optimal threshold of the assay yields the highest sensitivity relative to the specificity.

**Figure 1:**
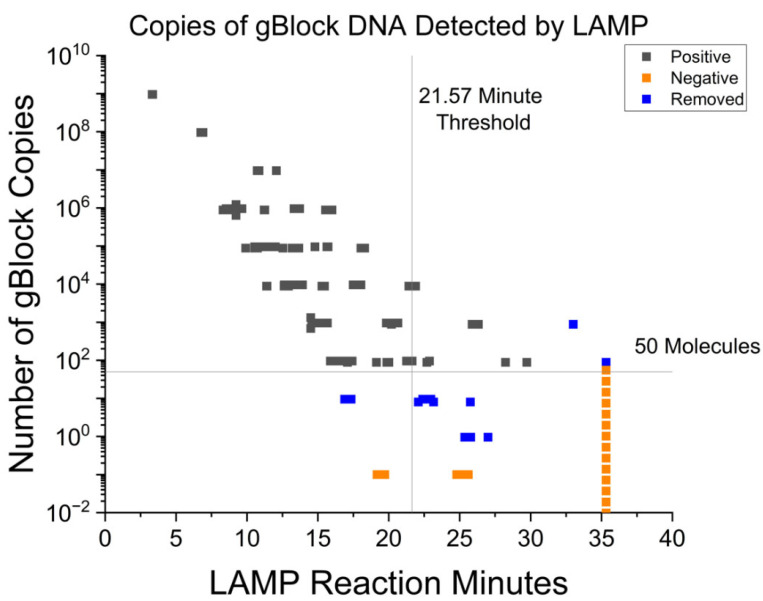
This figure shows the results of LAMP assays with spiked gBlock DNA of known quantities to generate a standard curve of the assay. The black squares are samples spiked with a known quantity of gBlock DNA, and were considered to be positive controls. The orange squares are samples that were not spiked with gBlock DNA, and were considered negative controls. The blue squares are samples that were determined as outside the limit of detection, and excluded in the data set, as described in the body of the manuscript.

The samples were analyzed by LAMP and data are show in in Figure 1. The threshold was determined by calculating the optimal balance of true positives to false positives. While comparing our time to amplification of the spiked 10^1^ samples, we saw that there were inconsistent time differences between spiked samples and samples not containing gBlock DNA. Thus, the assay is not sensitive enough to consistently detect fewer than 10 gene copies. The assay can reliably detect 88 copies or more of template. As such, values less than or equal to 10^1^ were excluded (as indicated by data represented in blue in Figure 1), and the statistic was re-evaluated. Using these parameters, the protocol had a limit of detection equal to or greater than 88 gene copies at 21.57 min, with an AUC of 0.97 (SE 0.01) with a 95% CI of 0.95 to 0.99, and a sensitivity of 91.5% and specificity of 90.5%. The ROC curve of clinical samples can be found in Supplementary Figure S1b.

### 3.2. Fluorescent RT-LAMP Limit of Detection in IAV either Grown in Cell Culutre or from Clinical Isolates

The next step was to examine the specificity and sensitivity of the M primers for M genes from viruses. To this end, nine different IAVs isolated from swine were propagated in cell culture and then tested in the LAMP reaction. The viruses were selected for their phylogenetic clade diversity (HA and NA), and were examined to have a representative sampling of currently circulating swine viruses (listed in Table 2). Viral genomes were isolated and RT-LAMP assays were performed in triplicate or quadruplicate, with 32 assays completed in total. For all nine viruses tested, the paired RT-qPCR Cq values were between 18 and 24. The RNA from all nine isolates was recognized by the M primers in the RT-LAMP assay, demonstrating that the primer set was effective at detecting various recent and relevant IAV-S isolates. Averaged RT-LAMP results and paired RT-PCR results for each isolate can be found in Table 3.

**Figure 2:**
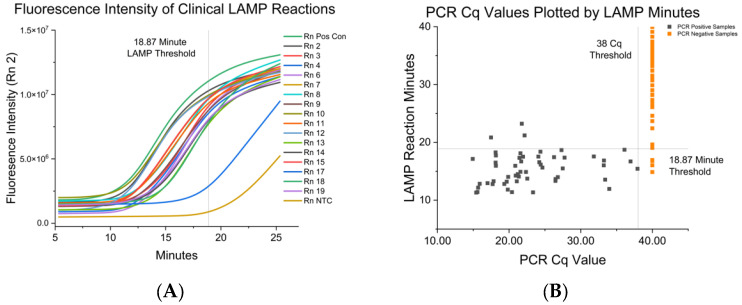
(**A**) This figure displays the change in fluorescence intensity measured over time during a clinical RT-LAMP assay. The top green line is the positive control, and the bottom gold line is the negative control. The 18.87 min threshold is displayed as determined by the ROC analysis. (**B**) This figure shows the results of paired clinical samples extracts as determined by LAMP and PCR. The black squares represent samples from PCR-positive pigs. The orange squares represent samples from PCR-negative pigs. The 18.87 min threshold for positive LAMP reaction was determined by ROC analysis. Samples that were positive in less than 18.87 min would be called positive by the LAMP assay.

To show that RT-LAMP could be used to detect IAV in clinical samples, we examined nasal swabs from pigs on four farms known to either have recent IAV infections or chronic mixed IAV infections (H1N1, H1N2, and H3N2 isolated on the farm since 2018). In total, samples from 72 pigs were collected and RNA was extracted from each sample. Paired RNA extracts were used for the RT-PCR and RT-LAMP assays. For samples screened by RT-PCR, it was found that 53 individual pigs (84 samples) tested positive for IAV while 19 individual pigs (96 samples) tested negative (Figure 2A). These assays were run on eight separate days to assess the reproducibility of the assay. The PCR values for the positive samples ranged from a Cq of 14–37. A sample was considered negative when the Cq value of >38, as determined by normalized RT-PCR data.

Interestingly, the M primers distinguished between positive and negative samples in the RT-LAMP assay from the paired RT-qPCR samples. Figure 2A is an example of the amplification curves of clinical samples and summarizes the fluorescence intensity of a LAMP reaction over time. In Figure 2B, the Cq value was plotted on the X-axis and compared to results from the paired RT-LAMP reaction. Several negative controls were detected under the previously determined threshold for synthetic DNA, so a new ROC analysis was carried out for the clinical samples, as performed previously. After analyzing the time of amplification (TOA) ROC data of the paired RT-LAMP samples, we determined that the assay had a 94. sensitivity and 94.85% specificity at a cutoff of 18.87 min. The area under the ROC curve (AUC) was 0.98 (SE 0.01) with a 95% CI of 95.65 to 100.0 with a calculated optimal assay cutoff of 18.87 min. The ROC curve of clinical samples can be found in Supplementary Figure S1a. This is in accordance with the gBlock DNA positive control assayed previously. Based upon these results, we can extrapolate that the assay is sensitive enough to detect 100 copies of matrix cDNA produced by the RT-LAMP reaction. Results are summarized in Table 4.

## 4. Discussion

We have developed an IAV-S RT-LAMP primer that detects the M gene from multiple US swine field isolates in less than 20 min of an assay run time. We began with validation of these primers using synthetic M genes. Then, we used a collection of nine isolates of IAV-S to show that the M primers can detect diverse IAV-S strains. After an initial lab validation and estimation of limit of detection, clinical samples were collected and assayed. We found that the RT-LAMP assay can rapidly detect clinical isolates, with similar sensitivity (94.34%) and specificity (94.85%) to the gold standard RT-PCR.

The WHO has prioritized eight viral diseases of public health importance, all of which are zoonotic or vector borne [40]. Diagnostic tests for zoonotic diseases in animals are being developed to rapidly identify these pathogens in different settings (e.g., farms or the wild). The 2009 influenza pandemic, which originated in swine in west-central Mexico, has spurred increased surveillance for IAV in commercial swine populations [41]. Currently, IAV is screened via RT-qPCR at centralized diagnostic laboratories because current point-of-care testing methods for swine on farms are much less sensitive than traditional RT-qPCR [42,43,44]. This current screening method is costly in terms of time and money. In contrast, RT-LAMP is a low-cost (USD 5 for extraction and LAMP reagents), low-overhead NAAT that can be easily performed with minimal lab equipment, and this makes it ideal to use as the basis for developing tests to detect the presence of viruses in agricultural or wild animals in field settings [14,45,46]. For influenza, the M gene is well conserved over the 825 sequences chosen, which included all clades and subtypes isolated from swine from 2017 to 2020. The sequences were analyzed and aligned for primer development, and only 15 out of 300 bases had less than 90% conserved identity in the primer target regions, making it a viable target for LAMP.

There are multiple approaches that were purposefully used to design a system for ease of use by swine producers in a farm setting. In commercial swine operations, for the detection of IAV-S in herds, oral fluid sample collection is often employed. Nasal swab samples were used for detecting the M gene here because the task of obtaining nasal swabs can be easily taught to swine producers. The extraction method for assay development used here included a magnetic tube rack and an adjustable heat block. These items are low cost and easy to use. The extraction kit reagents are stable at room temperature, reducing the need for cold-chain transportation and storage. There are still more opportunities to modify this assay to make it better for testing in the field. For example, there may be other extraction methods that reduce the time needed for the extraction of the sample prior to RT-LAMP [40,47]. Another step for the development of this assay in the field would be low-cost fluorescent readouts. Currently, there are devices designed for RT-LAMP use with a smartphone. Using the phone’s battery, the device powers a heat block and an LED, and uses the phone’s camera to detect the fluorescent emissions in real time [48].

## 5. Conclusions

In summary, we developed an RT-LAMP assay for the detection of the IAV-S matrix gene. The assay was successful in the detection of IAVs that were either grown in laboratory settings or from nasal swabs from infected pigs in farm settings. The assay’s high sensitivity and specificity suggest that this detection method is readily applicable for laboratory diagnostics. Further development of this assay for implementation in field settings would make this an important tool to aid in the rapid and low-cost diagnosis of IAV-S, which would help minimize or mitigate infections in farm settings.

## Figures and Tables

**Table 1:** RT-LAMP primers targeting the matrix gene.

Primer	Sequence (5’–3’)	Concentration in 10 × Mix (µM)	Matrix SequencePosition
F3	CAA GGA GGT GTC ACT AAG C	2	361–374
B3	CAT CTG CCT AGT CTG ATT AGC	2	641–661
FIP	GTG AGA CCG ATG CTG TGA ATC AGG AAC AGT GAC CAC AGA AG	16	496–511
BIP	ACC ACC AAT CCA CTA ATC AGG CGC CAT CTG TTC CAT AGC C	16	527–545
LoopF	TCT GTT CAC AAG TGG CAC A	4	461–485
LoopB	ACA GAA TGG TGC TGG CTA G	4	555–573

**Table 2:** This table contains the initial laboratory strain, and nine swine strains that were cultured and tested for LAMP assay efficacy. The strains were chosen based on the HA and NA sequence diversity, as listed here, and were classified using the ISU FLUture HA Sequence Identity Tool.

Subtype	Strain and Accession Numbers	H1/H3	N1/N2
H1N1	A/California/07/2009 641809.71	H1N1pdm09	Pandemic
H1N1	A/swine/Minnesota/A02245728/2020	Gamma	Classic
H1N1	A/swine/Iowa/A02479151/2020	Delta1a	Classic
H1N1	A/swine/Texas/A02245632/2020	Beta	Classic
H1N2	A/swine/Colorado/A02245414/2020	Delta1b	2002A
H1N2	A/swine/Indiana/A02478520/2019	Delta2	1998B
H1N2	A/swine/Iowa/A02478576/2019	Alpha	2002B
H3N2	A/swine/Nebraska/A02524799/2020	2010.1	2002B
H3N2	A/swine/Iowa/A02524878/2020	IV-A	2002B
H3N2	A/swine/NewYork/A01104005/2011	IV-A	2002A

**Table 3:** Summary of IAV-S isolate results for paired RT-PCR and RT-LAMP. The isolates showed similar time of amplification for all, showing the primer set is sensitive to many common field strains currently circulating in swine.

IAV-S Strain	RT-LAMP Mean and SD in Minutes to Positive	Paired RT-PCR Cq
H1N1 IA	12.85 ± 0.67	21.02
H1N1 MN	14.39 ± 0.62	19.50
H1N1 TX	12.66 ± 0.22	18.39
H1N2 CO	14.83 ± 0.71	19.92
H1N2 IA	12.83 ± 0.30	21.04
H1N2 IN	10.97 ± 0.12	20.90
H3N2 IA	11.85 ± 0.79	23.13
H3N2 NE	13.45 ± 0.88	19.99
H3N2 NY	10.24 ± 1.98	18.70

**Table 4:** Summary of clinical sample result statistics based on ROC curve analysis. Clinical sample cutoff time was shorter than synthetic DNA detection.

Test Cutoff Time	Sensitivity%	Specificity %	95% CI	Asym. Probability
<18.87 min	94.34	94.85	95.35–99.62	2.97 × 10^−22^

## Data Availability

Data can be found in the Illinois Data Bank repository (Supplementary Materials).

## Supplementary Materials

The consensus matrix sequence and data can be downloaded at: https://www.mdpi.com/article/10.3390/vetsci10030220/s1.
