# Supplementary material for: RT-LAMP as Diagnostic Tool for Influenza—A Virus Detection in Swine
Source: Vet Sci. 2023 Mar 13;10(3):220. doi: 10.3390/vetsci10030220 (PMC10051247; doi:10.3390/vetsci10030220)

Supplemental materials:

1. Sample information and processing

Clinical samples were taken on three separate farms. Samples were nasal swabs collected from each nare and placed in 0.5-1.0 mL of BHI broth in 2 mL cryotubes. Samples were held on ice for transport to the lab, and then stored at -80C within 5 hours after the time of collected and stored at -80C until processing. All samples were individually labeled with pig ID, date, and farm ID. Samples were held for a maximum of six month prior to processing. For processing, samples were thawed, and BHI was added to dilute the samples to a total of 1.8 mL, and vortexed for 1 minute. 140 uL of sample was used for RNA extraction. 500 uL of sample was aliquoted and frozen separately for future use if necessary. Extracted RNA samples were used for PCR and LAMP immediately.

1. RNA Extraction

RNA extraction from samples was performed using the Cytiva Sera-Xtracta Virus/Pathogen kit following the manufacturer’s instructions. The protocol is as follows. 140 uL of a sample was added to a tube containing the “binding/lysis reagent” and Proteinase K. The tube was incubated on a heat block at 60C for 10 min. Next, the tube was placed on a magnet stand for one minute and the supernatant was removed. The tube was removed from the magnet and the material remaining in the tube was washed with 950 ul of wash buffer 1. The tube was removed from the magnet and 80% ethanol was added as a second wash. The tube was placed back on the magnet stand for one minute and the supernatant was removed. The tubes were briefly removed and reapplied to the magnet stand to allow remaining ethanol to fall to the bottom of the tube, and the excess ethanol was removed. The tubes were then allowed to air dry for two minutes, The nucleic acids were eluted off the beads into 75C nuclease free water by gently mixing the sample with the water, and replacing the tube on the stand. No DNase or RNase treatment was used. It was assumed that the clinical samples would contain host DNA/RNA, as well as any commensal or environmental DNA/RNA. Thus, RNA purity or quantification was not assessed following the extraction, as this was not measurable without direct sequencing of samples.

1. PCR protocols

Two PCR protocols were used for verification of the LAMP assay. The first protocol is modified from the WHO information for the molecular detection of influenza viruses (7^th^ edition) and was used to detect synthetic matrix gBlock. Invitrogen SuperScript III Platinum SYBR green One-Step kits from Applied Biosciences (Waltham, MA) was used per manufacturer’s instructions. The primers used were as following, and taken from the WHO information for the molecular detection of influenza Viruses: Forward primer: M30F2/08 ATGAGYCTTYTAACCGAGGTCGAAACG, Reverse primer: M264R3/08 TGGACAAANCGTCTACGCTGCAG. Per reaction, 1 uL of SuperScript III, RT/Platinum TaqMix, 25 uL Sybr green mix, 1 uL Forward Primer, 1 uL Reverse primer, 0.1 uL of ROX dye, 17.15 uL DEPC treated water, and 4.75 ul of sample template was used. The kit used was a one-step kit, so reverse transcription evaluation was not performed. The PCR protocol was as follows: Reverse transcription was at 50C for 30 minutes, followed by PCR activated at 95C for 15 minutes. Denaturation was at 94C for 30 seconds, Annealing at 50C for 30 seconds, and extension at 72C for one minute. This was repeated for 45 cycles. Final extension was at 72C for 10 minutes. A melt curve cycle was evaluated after each run to ensure the quality of the reaction and rule out possible aberrant PCR products. The estimated amplicon size was 244 bp.

Clinical samples were evaluated with the VetMAX-Gold SIV Detection Kit, a USDA licensed, commercialized Real-time reverse transcription PCR assay for SIV RNA isolated from nasal swabs. This kit claims to be a qualitative kit, not a quantitative kit and uses primer-probe chemistry. This kit does not detect matrix gene, so we are unable to use it to compare the gBlock with clinical samples. The kit was used according to manufacturer's instructions. Per reaction, 12.5 uL 2x Multiplex RT-PCR buffer, 2.5 uL Multiplex RT-PCR enzyme mix, 1uL of Primer-Probe mix, 1 μL of Nuclease free water, and 8 μL of RNA template was used. The PCR method followed manufacturer’s instructions, and is as follows: Reverse transcription was at 48C for 10 minutes, followed by RT inactivation and initial denaturation at 95C for 10 minutes. Denaturation was at 95C for 15 seconds, followed by annealing and extension at 60C for 45 seconds. This was repeated for 40 cycles.

Figure S1. This is an ROC curve of the clinical (1a) and gBlock DNA samples (1b), showing the high sensitivity and specificity of the assay with the computed optimal cutoff point for each assay shown by the red circle.

1b

1a


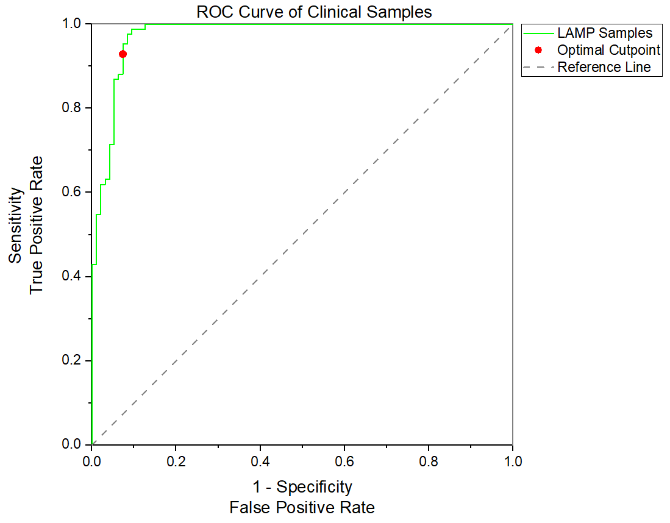

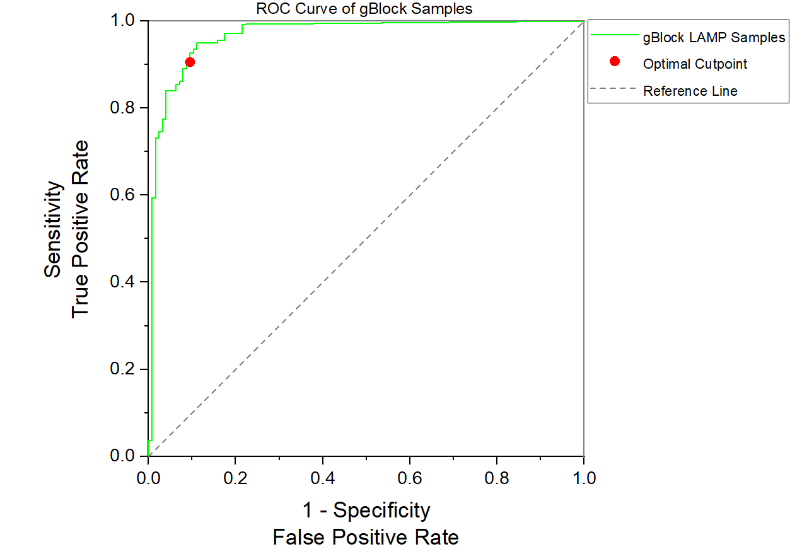

Supplement: Supplementary file 1 [file vetsci-10-00220-s001.zip › Supplementary materials 3-6.docx]
